# Night and day: Shrinking and swelling of stems of diverse mangrove species growing along environmental gradients

**DOI:** 10.1371/journal.pone.0221950

**Published:** 2019-09-03

**Authors:** Maria P. Vilas, Matthew P. Adams, Marilyn C. Ball, Jan-Olaf Meynecke, Nadia S. Santini, Andrew Swales, Catherine E. Lovelock

**Affiliations:** 1 School of Chemical Engineering, The University of Queensland, St Lucia, QLD, Australia; 2 CSIRO Agriculture and Food, Biosciences Precinct, St Lucia, QLD, Australia; 3 School of Earth and Environmental Sciences, The University of Queensland, St Lucia, QLD, Australia; 4 School of Biological Sciences, The University of Queensland, St Lucia, QLD, Australia; 5 Research School of Biology, Australian National University College of Science, Australian National University, Canberra ACT, Australia; 6 Griffith Centre for Coastal Management, Griffith University, Gold Coast, QLD, Australia; 7 Cátedra Consejo Nacional de Ciencia y Tecnología, Crédito Constructor, Benito Juárez, Ciudad de México, México; 8 Instituto de Ecología, Universidad Nacional Autónoma de México, Ciudad Universitaria, Ciudad de México, México; 9 National Institute of Water and Atmospheric Research, Hamilton, New Zealand; UNAM, MEXICO

## Abstract

Tree stems swell and shrink daily, which is thought to reflect changes in the volume of water within stem tissues. We observed these daily patterns using automatic dendrometer bands in a diverse group of mangrove species over five mangrove forests across Australia and New Caledonia. We found that mangrove stems swelled during the day and shrank at night. Maximum swelling was highly correlated with daily maxima in air temperature. Variation in soil salinity and levels of tidal inundation did not influence the timing of stem swelling over all species. Medium-term increases in stem circumference were highly sensitive to rainfall. We defoliated trees to assess the role of foliar transpiration in stem swelling and shrinking. Defoliated trees showed maintenance of the pattern of daytime swelling, indicating that processes other than canopy transpiration influence the temporary stem diameter increments, which could include thermal swelling of stems. More research is required to understand the processes contributing to stem shrinking and swelling. Automatic Dendrometer Bands could provide a useful tool for monitoring the response of mangroves to extreme climatic events as they provide high-frequency, long-term, and large-scale information on tree water status.

## Introduction

Mangrove forests are of major ecological and socio-economic importance for the functioning of coastal systems in the tropics and sub-tropics [1], and have been broadly recognised as a significant contributor of carbon sequestration to the global carbon balance [2,3]. However, global warming and associated rise in sea level are inducing changes in the structure, function and distribution of these forests. Higher temperature minima reducing exposure to frosts have been associated with poleward expansion of mangroves in salt marsh habitats near latitudinal limits [4]. In contrast, extreme temperature maxima coupled with drought have been associated with mangrove forest die-back, particularly along arid coastal systems [5–7]. The unprecedented die-back of 700 km of mangrove forest along the coast of the Gulf of Carpentaria in northern Australia during extreme El Niño conditions is anticipated to have far reaching effects on coastal ecology and commercial fisheries with unknown potentials for recovery [7]. This die-back of mangrove forest is consistent with a global pattern in forest die-back that has been increasing with the progression of climate change over the past decade, particularly in areas subject to combined effects of drought and extreme high temperatures [8]. The emergence of a global pattern in forest dieback underscores the importance of identifying both the causes of vulnerability and the mechanisms of tolerance. Much progress has been made in understanding some components of drought tolerance, such as hydraulic vulnerability of trees, including mangroves, while much less is known about the role of stem water storage [9]. Insights into the depletion and replenishment of stem water storage under varying climatic and environmental conditions are needed to better characterise the vulnerability of mangrove forests to climate change.

Stems of trees provide storage of water that can support photosynthetic carbon gain [10]. Over the time-scales of seasonal changes, stems of mangroves have been shown to progressively shrink during prolonged exposure to dry season conditions indicating depletion of stem water storage [11]. Stem swelling has been shown to occur in response to rainfall [12] consistent with both the rehydration of dehydrated stems and the opportunistic, patchy growth of wood [13,14]. Long-term changes in water storage are largely influenced by the environmental conditions [1]. Specifically, the capacity of stems to supply water to leaves can decline with increasing salinity, with subsequent increase in limitations to carbon gain. Thus, understanding how long-term changes in water storage respond to environmental conditions is essential to assess the resilience of mangroves to a changing climate.

Diel stem diameter variations have long been related to change in the water status of the stem, with shrinking occurring as stored water is exported to the canopy to meet demands imposed by high rates of transpiration during daylight, and subsequent nocturnal swelling and replenishment of water storage occurring when rates of canopy water loss are minimal [15–19]. Diel stem diameter variations have also been related to thermal swelling of the tissues and water within the tissues [10,20,21], although thermal swelling tends to be smaller than the swelling driven by stem re-hydration [22].

Recent studies in mangroves have revealed intriguing patterns in diel stem diameter variation. While, the expected pattern of daytime shrinking followed by nighttime swelling was observed in *Avicennia marina* growing in Queensland (Australia), a co-occurring mangrove species *Rhizophora stylosa* exhibited the opposite pattern [11]. Using a modelling approach, the unusual pattern of daytime stem swelling in *R*. *stylosa* was suggested to arise from osmotically driven change in stem storage tissue water potentials that drove radial movement of water into storage tissues while transpiration rates were high [23]. However, a different study found the unusual pattern of daytime swelling and nighttime shrinking to occur in stems of *A*. *marina* [24]. Using three-point dendrometers, strategically placed along the main stems, [25] found that in *A*. *marina* growing in New Zealand the daytime swelling originated in the bark and was decoupled from transpiration-dependent shrinking in the xylem. They interpreted this daytime swelling as a phloem-generated turgor signal. Further research of stem diameter variation can provide greater insights into the atypical pattern of daytime swelling in mangroves. Because trees can lose up to 99% of the stored water through foliar transpiration [26], studying the role of leaf transpiration in the atypical pattern of daytime swelling, for example by defoliating trees, is an important research topic.

Variations in the diel patterns of swelling and shrinking across different species could emerge due to interspecific differences in wood structure and mechanical properties that influence water transport [27]. For example, *A*. *marina* has successive cambia forming alternating layers of phloem and xylem [13,27]. However, species from the Rhizophoraceae, Meliaceae and other families exhibit typical secondary growth with internal xylem and external phloem. Additionally, species vary in their photosynthetic water use efficiency and thus their canopy water use [28], which may contribute to differences in the timing of stem swelling. Variations in the diel patterns of swelling and shrinking are to be expected across different environmental settings. In mangroves, the hydraulic conductivity declines with increasing salinity [29], which may also contribute to differences in the timing of stem swelling, although the daily patterns in stem swelling and shrinking in *A*. *marina* have shown no relationship to variations in soil water salinity [13]. Further study of stem diameter variation across different species and environments would provide greater insights into the causes of the atypical patterns of daytime swelling.

In the present study, we installed Automatic Dendrometer Bands at four mangrove forests in Australia and one in New Caledonia to capture responses of five species to variation in key environmental conditions. We evaluated the role of foliar transpiration on the timing and magnitude of swelling of stems by defoliating trees. This dataset enabled us to address the following five hypotheses: (1) mangrove species growing in similar environments differ in their diel patterns of stem swelling and shrinking, (2) environmental conditions alter the diel patterns of stem swelling and shrinking, (3) transpiration of leaves has a strong influence over daily patterns in swelling and shrinking, (4) the contribution of thermal swelling of water within stems to the diel changes in stem circumference is low relative to stem re-hydration, and (5) environmental conditions influence the medium-term patterns in stem swelling and shrinking.

## Materials and methods

Permission to conduct research at the Njimec mangrove forest of Northern Ouvéa was granted by the Téouta tribe. We were guided in the forest by Jean-Baptiste Dao, a guardian of the forest. Permission to conduct research in Giralia Bay was granted by the Department of Parks and Wildlife, Government of Western Australia (#SF010918). Permission to conduct research in Queensland was granted by the Department of National Parks, Sport and Racing, Government of Queensland (QS2017/CVL1330).

### Forests of study

Five mangrove forests across Australia and New Caledonia were studied (S1 Fig). The first forest is in Giralia Bay in the Exmouth Gulf (Western Australia, Australia). The Exmouth Gulf is an inverse estuary with infrequent river flows that occur during cyclones [30]. The mangrove forest is located on the edge of a tidal creek within Giralia Bay and is dominated by *A*. *marina*. The second forest is in a riverine setting within the South Arm of the Daintree River system (Queensland, Australia). This area is comprised of a high diversity of species. The third forest is in an estuarine setting along the Noosa River (Queensland, Australia). The Noosa River is comprised of shallow embayments connected by a river channel [12], and a sand bar in the mouth of the river, which restricts seawater exchange [31]. This forest in dominated by the species *A*. *marina*. The fourth forest is in Terranora Broadwater, an estuarine setting along the Tweed River (New South Wales, Australia). This location is comprised of a berm, which separates the water from the mangrove forest within a shallow basin [32]. This forest is also dominated by the species *A*. *marina*. The fifth forest is in Ouvéa Atoll (Loyality Islands, New Caledonia) and it is comprised of the species *Brugueira gymnorrhiza* and *R*. *stylosa*. The forests were chosen to cover different species (five species) and hydrological settings. For each forest, the dominant species were selected and therefore *A*. *marina* was more represented than other species. The hydrological setting and mean meteorological values (Australian Bureau of Meteorology, 2017) are summarized in Table 1.

**Table 1:** Characteristics of our forests of study including hydrological setting (riverine, estuarine or lagoon), mean annual rainfall, mean air temperature and soil porewater salinity.

Forest of study	Hydrological setting	Mean annual rainfall (mm y^-1^)	Mean air temp (°C)	Soil pore water salinity (ppt)
Noosa	Riverine	1,550	17–25	15–36
Terranora	Estuarine	1,700	16–25	18–28
Giralia Bay	Estuarine	260	18–32	42
Daintree River	Riverine	2,940	23–29	8
Ouvéa Atoll	Lagoon	1,684	24–29	35–36

### Dendrometer measurements

The experimental design implied the selection of eight distinct sites within our five forests of study to compare among different hydrological settings, species and defoliated versus non-defoliated trees (Table 2). We collected Automatic Dendrometer Bands (DBL60 stand-alone logging dendrometer, ICT International, Armadale, Australia) data at different times between February 2010 and July 2017. We installed one Automatic Dendrometer Band on straight sections of the main stem of each tree at breast height (1.3 m above the soil surface), or in the case of tall *Rhizophora* spp., on the straight section of the stem above the highest prop root. By installing the bands at the same height, we minimised potential effects arising from vertical variations in stem swelling and shrinking.

**Table 2:** Measurement details of trees assessed at each site within our five forests of study: Tree species, the number of trees of each species assessed, the dates of the measurements, the logging interval of the automatic dendrometer bands and the treatments.

Forest of study	Site	Geographic coordinates	Species	Number of trees measured	Time period assessed	Logging interval (min)	Treatment
Noosa River	Noosa Inner	26.31°S, 152.98°E	*A*. *marina*	3	21 Feb 2010–04 Mar 2011	60	Salinity gradient and medium-term environmental effects
	Noosa Outer	26.36°S, 153.04°E	*A*. *marina*	3			
Terranora	Terranora Lower	28.24°S, 153.50°E	*A*. *marina*	3	23 May 2011–30 Oct 2011	60	Salinity gradient and medium-term environmental effects
	Terranora Upper	28.22°S, 153.51°E	*A*. *marina*	3			
Giralia Bay	Giralia Lower	22.46°S 114.24°E	*A*. *marina*	8	14 Jul 2015–23 Jul 2015	30	Salinity gradient and defoliation
	Giralia Upper	22.46°S 114.24°E	*A*. *marina*	8			
Daintree River	Daintree River	16.32°S 145.41°E	*B*. *gymnorrhiza*	3	20 Aug 2015–01 Nov 2015	20	Species differences
		16.29°S 145.41°E	*R*. *apiculata*	3			
		16.32°S 145.41°E	*R*. *stylosa*	3			
		16.30°S 145.42°E	*X*. *granatum*	5	14 Jul 2017–16 Sep 2017	30	Defoliation
Ouvéa Atoll	Ouvéa Atoll	20.65° S, 166.56° E	*B*. *gymnorrhiza*	3	19 Feb 2017–12 Jul 2017	60	Species differences
		20.65° S, 166.56° E	*R*. *stylosa*	3			

In the Giralia Bay forest, Automatic Dendrometer Bands were installed in *A*. *marina* trees that were 3–7 m high at two distinct sites: the lower intertidal (n = 8) and the upper intertidal (n = 8). Measurements were collected between 14 and 23 July 2015. On 20 July 2015, we defoliated three trees by clipping all leaf bearing twigs and monitored them for three consecutive days as we did not have the capacity to measure canopy transpiration measurements for an extended period. In the Daintree River forest, we chose large (30 m high) individuals of *Rhizophora apiculata* that grew close to the confluence of the South Arm and the Daintree River. We also chose individuals of *B*. *gymnorrhiza*, *R*. *stylosa*, *R*. *apiculata*, and *Xylocarpus granatum* that were 5–10 m high and grew within the higher reaches of the South Arm of the River. Automatic Dendrometer Bands were installed in *B*. *gymnorrhiza* (n = 3), *R*. *apiculata* (n = 3), and *R*. *stylosa* (n = 3), between August 2015 and November 2015. Automatic Dendrometer Bands were also installed in *X*. *granatum* (n = 5), between 14 July 2017 and 19 September 2017. The *X*. *granatum* trees were defoliated on 17 October 2017 to assess the effect of transpiration on the timing of swelling and shrinking of stems. Defoliation of all five *X*. *granatum* trees was achieved by manually clipping all leaf bearing twigs off the trees. In the Noosa River forest, Automatic Dendrometer Bands were installed in *A*. *marina* trees that were 10–15 m high at two distinct sites: the inner estuarine site (n = 3) and the outer estuarine site (n = 3). The inner estuarine setting is located at Lake Cootharaba, 19 km away from the river mouth (Noosa Inner). The outer estuarine setting at Lake Cooroibah (Noosa Outer) is located 8 km away from the river mouth. Measurements were performed between February 2010 and March 2011. In the Terranora forest, Automatic Dendrometer Bands were installed in *A*. *marina* trees that were 10–15 m high at two distinct sites: an upper (n = 3) and a lower intertidal (n = 3) site. The upper intertidal site is located 50 m away from the shore, while the other site is located at a lower basin, 140 m away from the shore. Measurements were collected between May 2011 and October 2011. Finally, in the Ouvéa Atoll forest, Automatic Dendrometer Bands were installed on individuals of *R*. *stylosa* (n = 3) that were 5–10 m high and close to the mouth of the mangrove lagoon, as well as on tall (20 m high) *B*. *gymnorrhiza* (n = 3) under less saline conditions further within the mangrove lagoon. Measurements were performed between February 2017 and July 2017.

### Environmental data

To assess the effect of the environment on the changes in stem diameter, climate data for the Terranora and Noosa River forests were sourced from the Australian Bureau of Meteorology (http://www.bom.gov.au). Terranora and Noosa River were chosen as the time-series spanned different seasons. Rainfall (mm) data for Terranora were obtained from a station located approximately 5 km east of the study sites (station number: 58056), and solar exposure (MJ/m^2^) data were derived from satellite data for this station; both variables were available at daily intervals. Relative humidity (%), and air temperature (°C) were obtained from a station located approximately 7 km northeast of the study sites and were available at 3-hour intervals (station number: 40717). Tidal inundation (m) data for this station were obtained from the NSW Office of Environment and Heritage (http://mhl.nsw.gov.au). The tidal gauge was located approximately 3 km north of the Terranora sites and tidal data was available at 15 min intervals. Meteorological data for Noosa were obtained from a station located approximately 5 km south of Noosa Outer site and approximately 10 km south of Noosa Inner site (station number: 40908). Vapour pressure deficit (VPD, kPa) was calculated from relative humidity and air temperature data [33].

### Data analyses

#### Diel changes in stem shrinking and swelling

Stem diameter variation data were first scanned and corrected for band adjustments and spikes in the data. Dendrometer data that changed by more than 0.35 mm within one hour were considered unrealistic and eliminated from the analysis. Subsequent measurements were also removed until visual inspection indicated that the sensor signal was showing a similar pattern than that before the position change. All trees with less than 50% of the data remaining after the corrections were excluded from the analysis (1 tree from Giralia Bay). Since Automatic Dendrometer Bands were at times exposed to direct sunlight that could have affected the diel changes in stem circumference, raw Automatic Dendrometer Bands measurements were corrected for temperature expansion of the band following [11]. The circumference changes since the start of the experiment (Δc, mm) were calculated by subtracting the Automatic Dendrometer Band measurements from the initial Automatic Dendrometer Band value at time zero [15]. The residual variation (Δc_r_, mm) was the difference between the Automatic Dendrometer Bands measurements and the smooth spline fitted to daily averaged dendrometer data [15]. The magnitude of daily stem swelling was characterised by the maximum Δc_r_ [15]. The times of maximum swelling and shrinking were estimated as the times at which the daily maximum and minimum in Δc_r_ were attained, respectively. These analyses were performed using self-build functions in Matlab R2014b (The MathWorks, Inc., Natick, Massachussets, USA).

We used analysis of variance (ANOVA) to assess variation in the time of the day at which stems attained maximum swelling among: (1) species at the Daintree River forest (*B*. *gymnorrhiza*, *R*. *apiculata*, and *R*. *stylosa*) and species at the Ouvéa Atoll forest (*B*. *gymnorrhiza* and *R*. *stylosa*), (2) the six sites where *A*. *marina* was assessed (Noosa Inner, Noosa Outer, Terranora Lower, Terranora Upper, Giralia Bay Lower and Giralia Upper), and (3) *X*. *granatum* (Daintree) and *A*. *marina* (Giralia Bay) trees before and after defoliation. In this case, both the raw Automatic Dendrometer Bands data and the Automatic Dendrometer Bands data corrected for band temperature expansion were analyzed, because after defoliation the bands were exposed to higher levels of direct sunlight and thus potentially a different temperature environment. In addition, we assumed that transpiration occurs during the day, even if there can be slightly reduced transpiration at midday [28]. ANOVA was conducted using DataDesk 8.0 (Ithaca, New York).

In addition, the importance of thermal swelling relative to stem re-hydration (hypothesis 4) was assessed by estimating the expansion of water within stems. Specifically, for a stem with 10 cm diameter (circumference of 31.4 cm) we estimated lateral expansion for a 1 cm thick disc of stem that contained between 40–70% water (e.g. [34] in a range of temperate tree species). We calculated the expansion of the volume of water in the stem assuming that water expands at a rate of 0.00021 L per°C [35] and divided the results by two to account for both vertical and lateral expansion (equal vertical and lateral expansion was assumed). We then added this to the initial volume before recalculating the change in circumference of the stem.

#### Medium-term changes in stem shrinking and swelling

To distinguish diel stem circumference changes from medium-term circumference changes, a growth line was constructed by plotting a horizontal line between the first measurement of stem circumference and the next greater stem circumference value. This procedure was repeated until the last measurement. Thus, zero growth was assumed during periods of stem shrinking. This approach was applied because little growth occurs during periods of stem shrinking [36]. Stem water deficit (ΔW, mm) was calculated as the difference between the Automatic Dendrometer Band measurements and the growth line, which follows the maximum stem circumference values, therefore resulting in zero or negative ΔW values. ΔW was calculated only for sites where medium-term Automatic Dendrometer Bands measurements and meteorological data were available (Terranora and Noosa River forests).

A moving correlation analysis was then used to investigate medium-term changes in the relationships between single meteorological variables (rainfall, relative humidity, temperature and VPD) and the tree water deficit indicator ΔW at a daily resolution at the Terranora and Noosa River sites (hypothesis 5). Moving correlation analysis estimates temporal changes in these relationships by progressively shifting the period of a fixed number of days across time to compute the correlation coefficients. The length of the moving window was set to 30 days to provide robust measures of association between the variables [37] and the analysis was performed over 12 months at the Noosa River sites and 4 months at the Terranora sites. Since most variables were non-normally distributed, a Spearman rank-correlation coefficient was used. The strength of the correlation between meteorological variables and ΔW was assessed by calculating the percentage of time with significant correlation. With Terranora data, where rainfall was limited during the assessment, we further explored the link between air temperature, solar radiation and the variation in stem circumference. All these analyses were conducted in Matlab R2014b (The MathWorks, Inc., Natick, Massachussets, USA).

ANOVA was also used to assess variation in stem water deficit between sites at the Noosa River and Terranora. The relationship between solar radiation and variation in stem circumference was only studied at Terranora for comparison between trees present in the lower and upper intertidal zones and was assessed through analysis of covariance. For this analysis, only periods with no rain were chosen to reduce confounding factors. ANOVA and analysis of covariance were conducted using DataDesk 8.0 (Ithaca, New York).

## Results

### Stems of mangrove trees swell during the day

**Fig 1:**
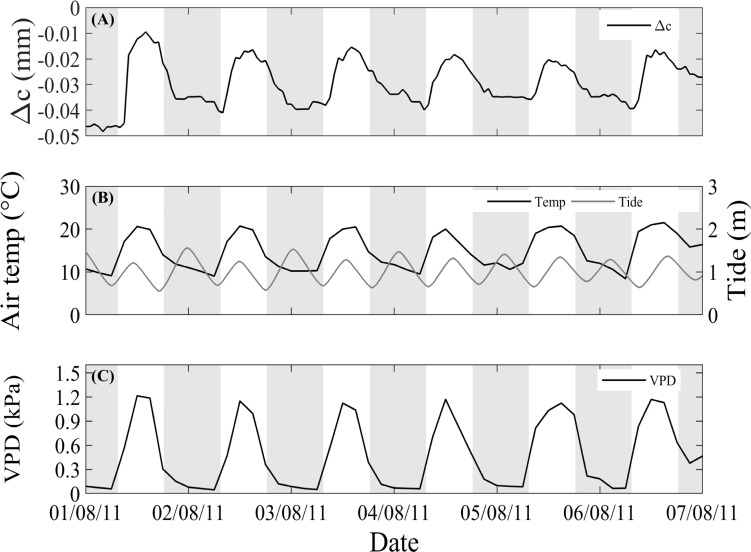
Typical stem circumference variation (Δc, mm) for *A*. *marina* using automatic dendrometer bands in a period of time with no rainfall. (A) Circumference variation (Δc, mm) measured at Terranora Upper, and (B) air temperature (°C) data from a meteorological station ~7 km northeast of Terranora study sites, and tidal height data from a station ~3 km north of Terranora study sites (m). (C) Vapour pressure deficit (VPD, kPA). The shaded areas in (a) and (b) indicate night-time.

**Fig 2:**
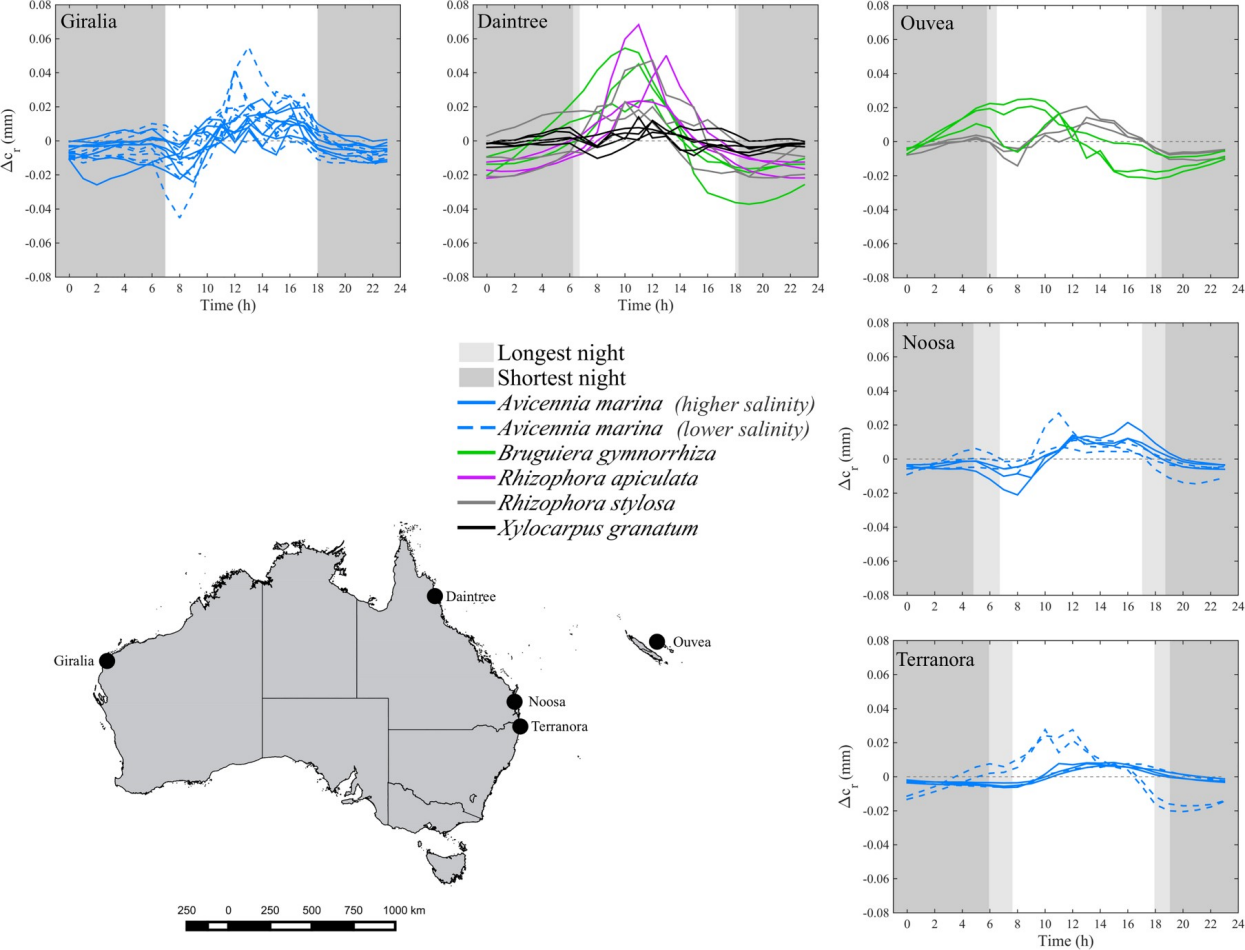
Mean daily patterns in residual variation in stem circumference (Δc_r_, mm) of mangrove trees over five forests (black circles). Forests are Giralia Bay (Western Australia), Daintree River (Queensland), Ouvéa Atoll (New Caledonia), Noosa (Queensland), and Terranora (Queensland). Different species included *A*. *marina* (blue), *B*. *gymnorrhiza* (green), *R*. *apiculata* (pink), *R*. *stylosa* (grey), and *X*. *granatum* (black). At some locations, stems were measured at multiple sites. At Giralia Bay and Terranora, *A*. *marina* trees growing in the more saline site are represented with blue dashed lines while trees in the less saline site are represented by blue solid lines. At Noosa, *A*. *marina* trees growing in the more saline site (outer estuarine) are represented by blue dashed lines, and trees from the less saline site (inner estuarine) by blue solid lines. The light grey shaded areas indicate the longest night, and the dark grey shaded areas indicate the shortest night, during the period of analyses.

All mangroves exhibited diurnal stem swelling. Maximum circumference typically occurred during the day, when air temperatures and VPD were highest (Fig 1A–1C). Tidal inundation usually showed two peaks in a period of 24 h, with one peak occurring during the day and the other during the night (Fig 1B). However, the daily change in stem circumference over all sites was not influenced by tidal inundation. Instead, an increase in stem circumference occured during the morning and a decrease in the afternoon, which closely tracks variation in air temperature and VPD (Fig 1 and Fig 2).

**Fig 3:**
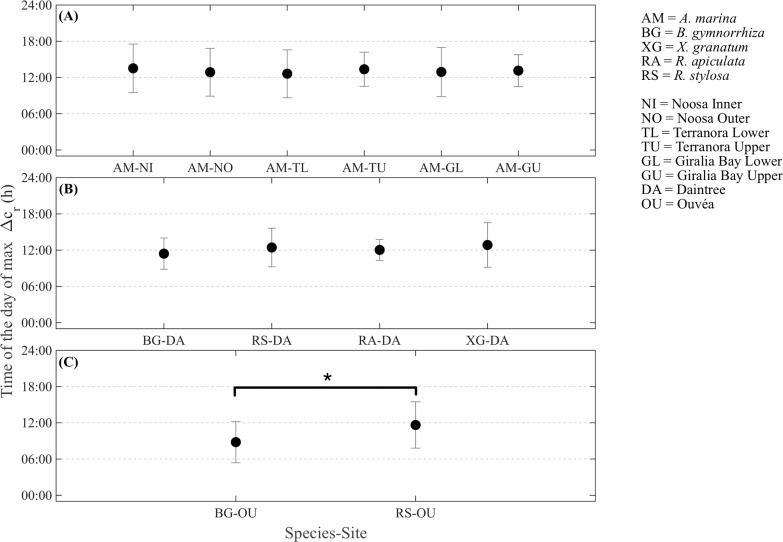
Mean and standard deviation of the time (h) at which the daily maximum residual variation (Δc_r_) occurred among: (A) *A*. *marina* stems over a range of sites, (B) Different species at the Daintree River, and (C) different species at Ouvéa Atoll. The dashed lines show 06:00, 12:00 and 18:00 h to facilitate the interpretation of the data. The asterisk in (C) indicates significant differences between the timing of maximum stem swelling between species at Ouvéa Atoll (BG-OU and RS-OU, (p < 0.05).

All stems of *A*. *marina* that were assessed showed maximum swelling in the early afternoon ranging from 12:37 ± 3:58 h to 13:31 ± 4:02 h (Fig 3A). The timing of maximum stem swelling of *A*. *marina* was not significantly different among the six sites where this species was assessed (F_(3,18)_ = 0.145, p = 0.929). Soil porewater salinity, which varied among sites at Noosa, and position in the intertidal, which varied at Terranora and Giralia Bay, had no significant influence on the timing of maximum swelling (Noosa F_(1,4)_ = 1.23, p = 0.330; Terranora _F(1,4)_ = 0.241, p = 0.649; Giralia Bay F_(1,14)_ = 0.061, p = 0.808). Although not statistically significant, trees located in the lower intertidal generally attained higher Δc_r_ values than those located in the upper intertidal (Giralia Bay and Terranora, see Fig 2). However, differences in Δc_r_ were not clearly observable in trees located along a salinity gradient at the Noosa River. In the Daintree River, *B*. *gymnorrhiza* stems attained maximum circumference earlier in the day (11:25 ± 2:35 h) compared with *R*. *stylosa* (12:26 ± 3:12 h), *R*. *apiculata* (12:02 ± 1:42 h) or *X*. *granatum* (12.90 ± 1:33 h) (Fig 3B); however, these differences were not significant (F_(3,9)_ = 0.903, p = 0.476). In Ouvéa Atoll, stems of *B*. *gymnorrhiza* expanded earlier in the day (8:47 ± 3.41 h) compared with stems of *R*. *stylosa* which reached maximum circumferences at approximately noon (F_1,4_ = 54.4; p = 0.0018) (Fig 3C).

Using a simplified model that accounts for the thermal swelling of water within stems, we estimated that stem circumference swelling could be between 0.003 and 0.195 mm for a variation in temperature of 0.5 and 15°C, respectively (see S2 Fig), which is within the range of our observations for Δc (Fig 1).

### Defoliated trees continued to swell and shrink

**Fig 4:**
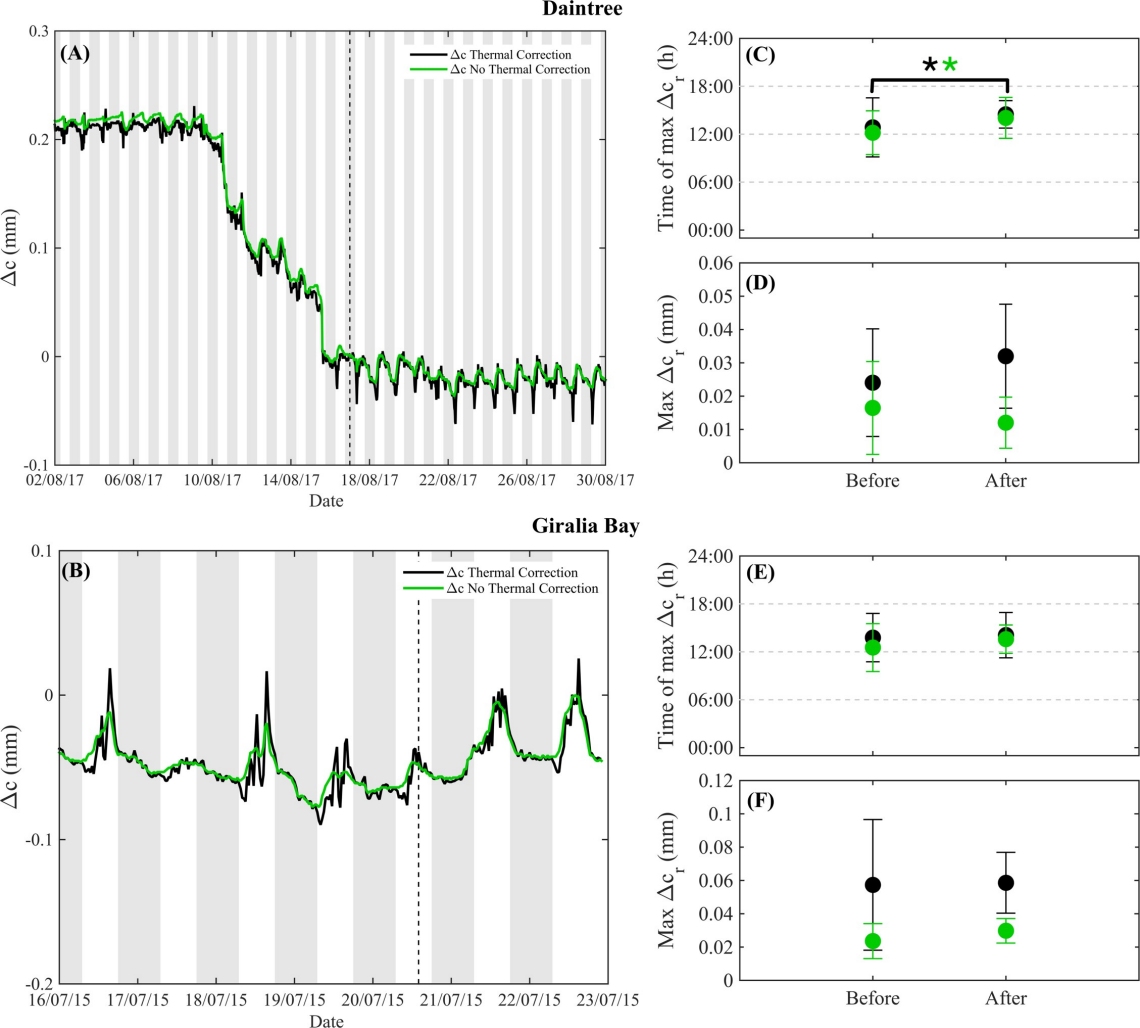
(A) Typical circumference variation (Δc, mm) pattern before and after defoliation of stems of *Xylocarpus granatum* from the Daintree River and (B) *A*. *marina* from Giralia Bay. (C) Mean and standard deviation of the time at which the daily maximum residual variation (Δc_r_) in stem circumference occurred before and after defoliation, and (D) mean and standard deviation of the maximum Δc_r_ before and after defoliation for *X*. *granatum* from the Daintree River. (E) Mean and standard deviation of the time of maximum Δc_r_ before and after defoliation, and (F) mean and standard deviation of the maximum Δc_r_ before and after defoliation for *A*. *marina* from Giralia Bay. The analyses with and without thermal correction of the band are shown in black and green, respectively. The shaded areas in (A) and (B) indicate nighttime. The vertical dashed lines in (A) and (B) indicate the defoliation date. The horizontal dashed lines in (C) and (E) represent the time of sunrise, midday and sunset. The two asterisks in (C) indicate that there was a significant difference between before and after defoliation regardless whether the thermal correction was applied or not (p < 0.05).

Both defoliated *A*. *marina* and *X*. *granatum* stems continued to expand during the day and contract during the night (Fig 4A and 4B), both with and without applying a thermal correction for the band, although thermal correction of the band caused more variability in the data (Fig 4). For *X*. *granatum*, where our record after defoliation was longer, defoliation was associated with stabilisation of stem circumference, which had been dropping through the dry season since bands were attached (Fig 4A). After defoliation the mean timing of maximum stem swelling significantly progressed by about one hour (from ~13:00 h to ~14:00 h) over the 30 days we monitored stems after defoliation, irrespective of whether we applied a thermal correction for the band (F_1,8_ = 5.36. p = 0.0492) (Fig 4C). The maximum residual stem circumference variation was higher when a thermal correction for the band was applied (Fig 4D), but there were no significant differences between before and after defoliation irrespective of whether the band thermal correction was applied, suggesting that the use of thermal correction for the band is unlikely to have influenced our results.

### Changes in water storage are highly sensitive to rainfall

**Fig 5:**
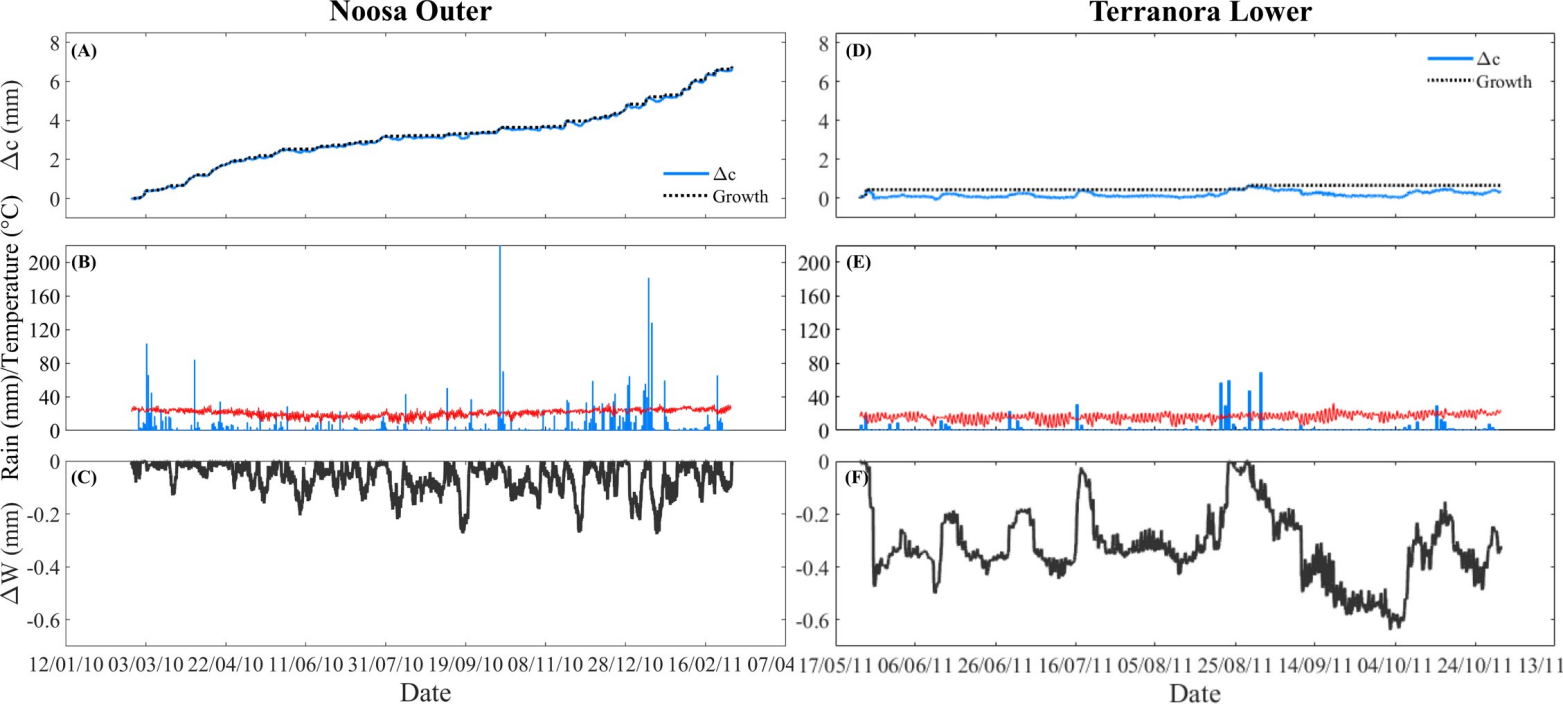
Typical time course of the increment in stem circumference (Δc, mm) for *A*. *marina* trees at (A-C) Noosa Outer and (D-F) Terranora Lower. Plots (A) and (D) show stem circumference change (Δc, mm, blue line) and growth line (mm, dashed line). Plot (B) and (E) show variation in rainfall (mm) and air temperature (°C), and plot (C) and (F) show the stem water deficit (ΔW, mm) which was calculated as the difference between the growth line and the daily band measurements (see Materials and Methods).

Changes in stem circumference over longer time periods (weeks to months) were highly sensitive to rainfall. While frequent rainfall events occurred at the Noosa River (57% of days), these were less frequent at Terranora, occurring in only 27% of days (Fig 5B and 5E). Daily rainfall records indicated maximum daily rainfall of 210 mm at the Noosa River and 70 mm at Terranora, which were coincident with large increases in stem circumference and low stem water deficits (ΔW). Over one year, stem water deficit (ΔW) values of -0.27 mm (Noosa River) and -0.63 mm (Terranora) were typically observed in periods of low frequency rainfall or low levels of rainfall (Fig 5C and 5F).

**Fig 6:**
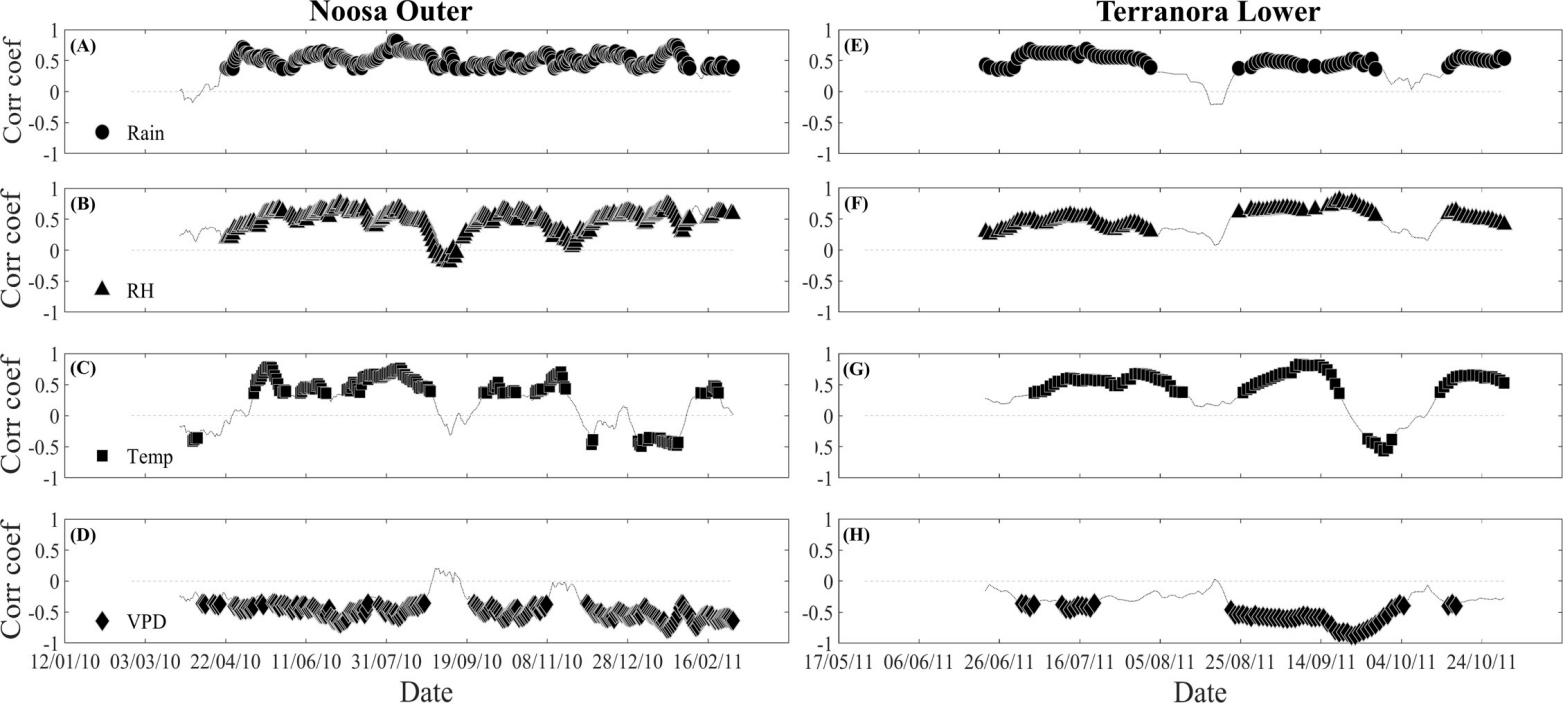
Typical moving correlation analysis (MCA, moving window = 30 days) between stem water deficit (ΔW) and environmental variables of *A*. *marina* trees from (A-D) Noosa Outer and (E-H) Terranora Lower sites. Plots show the results of moving correlation analysis between ΔW and (A and E) rainfall (rain, circle), (B and F) relative humidity (RH, triangle), (C and G) mean air temperature (Temp, square), and (D and H) vapour pressure deficit (VPD, diamond). A one-day lag was considered in calculating the correlation between rainfall and ΔW. Lines with no markers indicate non-significant correlations. The dotted line indicates no correlation (Corr coef = 0).

In general, the calculated ΔW showed a positive correlation with rainfall, relative humidity and air temperature, and was negatively correlated with VPD at both the Noosa River and Terranora forests (Fig 6 and Table 3). At the Noosa River, where rainfall was frequent, ΔW was significantly correlated with rainfall in more than 70% of days at both sites (71% and 76% of days at the outer and inner estuarine site, respectively). At the lower intertidal site at Terranora, ΔW was also most strongly correlated with rainfall (62% of days) and relative humidity (67% of days). At the upper intertidal site in Terranora, the moving correlation analysis revealed a lower sensitivity of ΔW to rainfall, relative humidity and VPD compared with the Noosa River sites and the lower intertidal sites at Terranora (Table 3). While the correlation between ΔW and air temperature was significant in 31–33% of days at the Noosa River, it was significant in 59–69% of days at Terranora (Fig 6C and 6G and Table 3). We also explored the role of solar radiation on the variation in stem circumference at Terranora. At this forest, stem circumference significantly increased with increasing daily solar radiation, although this was most apparent in the low intertidal and the relationship was highly variable (S3 Fig).

**Table 3:** Percentage of the automatic dendrometer band measurement period in which the correlation between stem water deficit (ΔW) and environmental variables was significant (p-value ≤ 0.05) and positive (rainfall, relative humidity, and air temperature) or significant (p-value ≤ 0.05) and negative (VPD). Correlations were assessed using a Spearman moving correlation analysis (MCA). A one-day lag was considered in the correlation between rainfall and ΔW.

	Percentage (range)			
	Noosa Outer	Noosa Inner	Terranora Lower	Terranora Upper
**Rainfall**	71% (0.36–0.82)	76% (0.36–0.79)	62% (0.36 − 0.70)	19% (0.36 − 0.55)
**Relative humidity**	62% (0.36–0.77)	56% (0.36–0.77)	67% (0.36 − 0.80)	46% (0.36 − 0.73)
**Air temperature**	33% (0.36–0.87)	31% (0.36–0.84)	59% (0.36 − 0.87)	69% (0.36 − 0.91)
**VPD**	52% (-0.77 − -0.36)	43% (-0.81 − -0.36)	48% (-0.87 − -0.36)	19% (-0.78 − -0.36)

## Discussion

### Swelling of all mangrove species occurs during the day

In this study, all mangrove species showed a pattern of daytime stem swelling across all sites. Mangrove stems typically began to swell in the morning and reached maximum circumference close to midday when air temperatures were maximal. Daytime swelling of stems has been previously observed in *R*. *stylosa* [11] and *A*. *marina* [24,25]. This diel pattern of daytime swelling and overnight shrinking is not the same as that observed in other studies of *A*. *marina* [11,13] or in other trees in which maximum shrinking was typically observed during the day when it is proposed that transpiration from the canopy results in depletion of stem water storage [10,15,38]. Previous studies suggest that the atypical pattern of daytime swelling may be a result of endogenous osmotic adjustment of elastic water storage tissues through the synthesis of sugars [23,24]. Indeed, [25] have identified bark as the source of daytime stem swelling and showed environmental effects on daytime swelling which could reconcile differences between their observations on *A*. *marina* with those of [14]. More research is needed to better understand the mechanisms underpinning differences in patterns of the timing of stem swelling and shrinking. This includes structural analyses of stem traits (such as the proportions of phloem and xylem) which vary across species and influence stem water content and stem water storage characteristics, together with explicit measurements of the relative contributions of different tissues to daily net changes in stem circumference.

We did not observe differences in the timing of stem swelling across species, except for Ouvéa Atoll, where *B*. *gymnorrhiza* attained maximum circumference hours before *R*. *stylosa*. Architectural and physiological differences between these two species [39] may be driving the difference in the timing of expansion. However, their synchronous swelling in the Daintree River suggests that other mechanisms are responsible for the differences at Ouvéa Atoll, which could include radial or vertical variations in properties related to density and water content [34,40]. It has been suggested that breast height measurements are usually not representative of the whole tree stem [41], thus further investigations using Automatic Dendrometer Bands at different stem heights need to be undertaken.

Despite a wide variation in soil porewater salinity (8–42 ppt) over the study sites, salinity at the roots did not appear to influence the timing or magnitude of stem swelling or shrinking, in line with previous observations in *A*. *marina* [13]. However, the higher intertidal site at Terranorra had slightly lower daily increments in stem circumference than trees growing lower in the intertidal zone, suggesting rooting environment may have an influence on stem water storage. Extending our study to trees that experience highly saline conditions may provide additional insights. Overall, these findings do not support our hypotheses that there would be interspecific or environmentally driven differences in the diel patterns of stem swelling and shrinking.

### The role of transpiration in stem swelling and shrinking

We found that all defoliated trees continued to swell and shrink during the days after defoliation. In the Daintree River, where we had a longer record pre- and post-defoliation, the steady shrinking of the stems during the dry season was curtailed after defoliation, but daily swelling and shrinking continued up to 30 days after defoliation when the bands were removed. In addition, no significant differences in the magnitude of stem swelling were observed between stem increments before and after defoliation. These observations do not support our third hypothesis that the patterns of daytime swelling and overnight shrinking are solely due to variation in plant water use.

Water uptake processes, involving for example aquaporins which are controlled by a circadian clock [42], may contribute to the continued post-defoliation oscillations in stem circumference. An alternative explanation, supported by our modelling approach, is that thermal expansion of water within the stems may be sufficient to account for the continued daily variation in stem circumference (Supporting information S2 Fig). Hence, while medium-term measures of stem circumference (positive or negative) can provide valuable information on growth and stem water storage, shorter term measurements may be highly influenced by thermal effects on water within stems. This requires further investigation.

The present study does not negate suggestions of previous authors [11,23–25] that daytime stem swelling concurrent with high canopy transpiration rates may reflect osmotically-driven radial transfer of water into living cells of stem tissues. However, such biologically driven sources of stem circumference variation would occur against a background of thermal swelling and shrinking in response to diel variation in temperature. The dendrometer band measurements used both previously [11,23,24] and in the present study did not distinguish between biological and thermal sources of stem swelling and shrinking, and all studies reported that diel patterns in stem circumference variation were correlated with temperature. We showed experimentally that defoliated trees continued to exhibit a pattern of stem circumference variation that was consistent in both timing and magnitude with thermal effects (Fig 4), the implication being that biologically driven effects may have been small relative to those due to temperature. More research is needed to better understand the relative contributions and interplay of thermal and biological effects to dynamic stem circumference variation. For instance, imaging techniques could be used to assess relative contributions of different tissues to dynamic variation in stem diameter [43].

### Environmental control of medium-term radial trends

Stem expansion occurred in response to rainfall events, supporting previous reports (and our fifth hypothesis) showing that changes in water storage in mangroves are closely linked to rainfall [11–14]. Stem expansion in response to rainfall has also been documented in other tree species [15,37,44]. Access to rainfall may come from preferential uptake of freshwater by roots at the soil surface [45] or through uptake by foliage [14,46,47].

In addition to rainfall, air temperature was highly and positively correlated with stem water deficit, highlighting the importance of air temperature in driving stem expansion. These results are different to those from other studies in which air temperature was commonly negatively correlated with stem water deficit [36,37]. In our study, a negative correlation between stem water deficit and temperature was only observed during periods of high rainfall, suggesting that rainfall may have masked the effect of temperature on stem water deficit. Air temperature could have direct effects via thermal swelling/shrinking of water within the tissues and indirect effects via the influence of VPD on evaporation and the water status of the stems [25]. Tree water deficits were also positively correlated with relative humidity in both the Noosa River and Terranora. Air humidity has been suggested as a driver of circumference change in trees [15]. These observations are consistent with previous studies showing that mangrove stems contract during periods of scarce rainfall, particularly on days of high VPD [11]. Overall, our results suggest that medium-term changes in stem water storage by mangroves is strongly dependent on rainfall, but that atmospheric humidity may also influence stem water storage.

In some sites, the Automatic Dendrometer Bands indicated that stems were shrinking over dry periods, as was also observed by [11,13]. Medium-term shrinking indicates a reduction in stem water storage, despite regular tidal inundation, underscoring the importance of rainfall for sustained mangrove productivity [48]. Although tree mortality was not observed in our current data set, medium-term deployments of Automatic Dendrometer Bands may provide useful indicators of the severity of water stress. For instance, decreasing radial growth has been suggested as early warning-signals of tree mortality [49]. Thus, linking Automatic Dendrometer Bands trends in stem water storage with loss of foliage, and/or branch and tree mortality, for example those that occurred in northern Australia [5,7], may provide quantitative, predictive assessments of the likelihood of partial or full mortality during extreme climatic events.

## Conclusions

Analyses of data from Automatic Dendrometer Bands are useful to understand the water status of mangroves, with long-term declining stem circumferences being indicative of persistent loss of stored stem water and increases indicating stem growth. Daily variations in stem circumference were observed, but defoliation experiments indicate this is potentially due to thermal swelling and shrinking of stem water. A useful pathway for future research is to discover the limits to mangrove stem water loss before morphological changes such as canopy loss begin to be observed. Development of a quantitative understanding of how the proportion of stem water in mangrove trees changes in response to foliage loss, branch death and shedding, and tree death, would provide valuable knowledge of the resilience of mangroves in the face of extreme climatic events.

## Supporting information

S1 Table Acronyms. (DOCX)

S1 Fig Mangrove forests. (TIFF)

S2 Fig Modelled thermal expansion of stems. Calculated potential variation in stem circumference with variation in temperature of the stem for a range of stem water contents and for temperature variations of 0.5–15°C.
(TIFF)

S3 Fig Stem circumference change vs. solar exposure. Residual variation (Δc_r_, mm) versus solar exposure (MJ/m^2^).
(TIFF)
