# Supplementary material for: Night and day: Shrinking and swelling of stems of diverse mangrove species growing along environmental gradients
Source: PLoS One. 2019 Sep 3;14(9):e0221950. doi: 10.1371/journal.pone.0221950 (PMC6719867; doi:10.1371/journal.pone.0221950)
Supplement: S1 Table — (DOCX) [file pone.0221950.s001.docx]

| **Description** | **Symbol** | **Calculated following** |
| --- | --- | --- |
| Circumference changes since the start of the experiment | Δc | Herrmann *et al.* (2016) |
| Residual variation | Δc_r_ | Herrmann *et al.* (2016) |
| Stem water deficit | ΔW | Ehrenberger *et al.* (2012) |

# References

Ehrenberger W., Rüger S., Fitzke R., Vollenweider P., Günthardt-Goerg M., Kuster T., … Arend M. (2012) Concomitant dendrometer and leaf patch pressure probe measurements reveal the effect of microclimate and soil moisture on diurnal stem water and leaf turgor variations in young oak trees. *Functional Plant Biology* **39**, 297–305.

Herrmann V., McMahon S.M., Detto M., Lutz J.A., Davies S.J., Chang-Yang C.-H. & Anderson-Teixeira K.J. (2016) Tree Circumference Dynamics in Four Forests Characterized Using Automated Dendrometer Bands. *PLOS ONE* **11**, e0169020.
