# Supplementary figures and images for: Night and day: Shrinking and swelling of stems of diverse mangrove species growing along environmental gradients
Source: PLoS One. 2019 Sep 3;14(9):e0221950. doi: 10.1371/journal.pone.0221950 (PMC6719867; doi:10.1371/journal.pone.0221950)

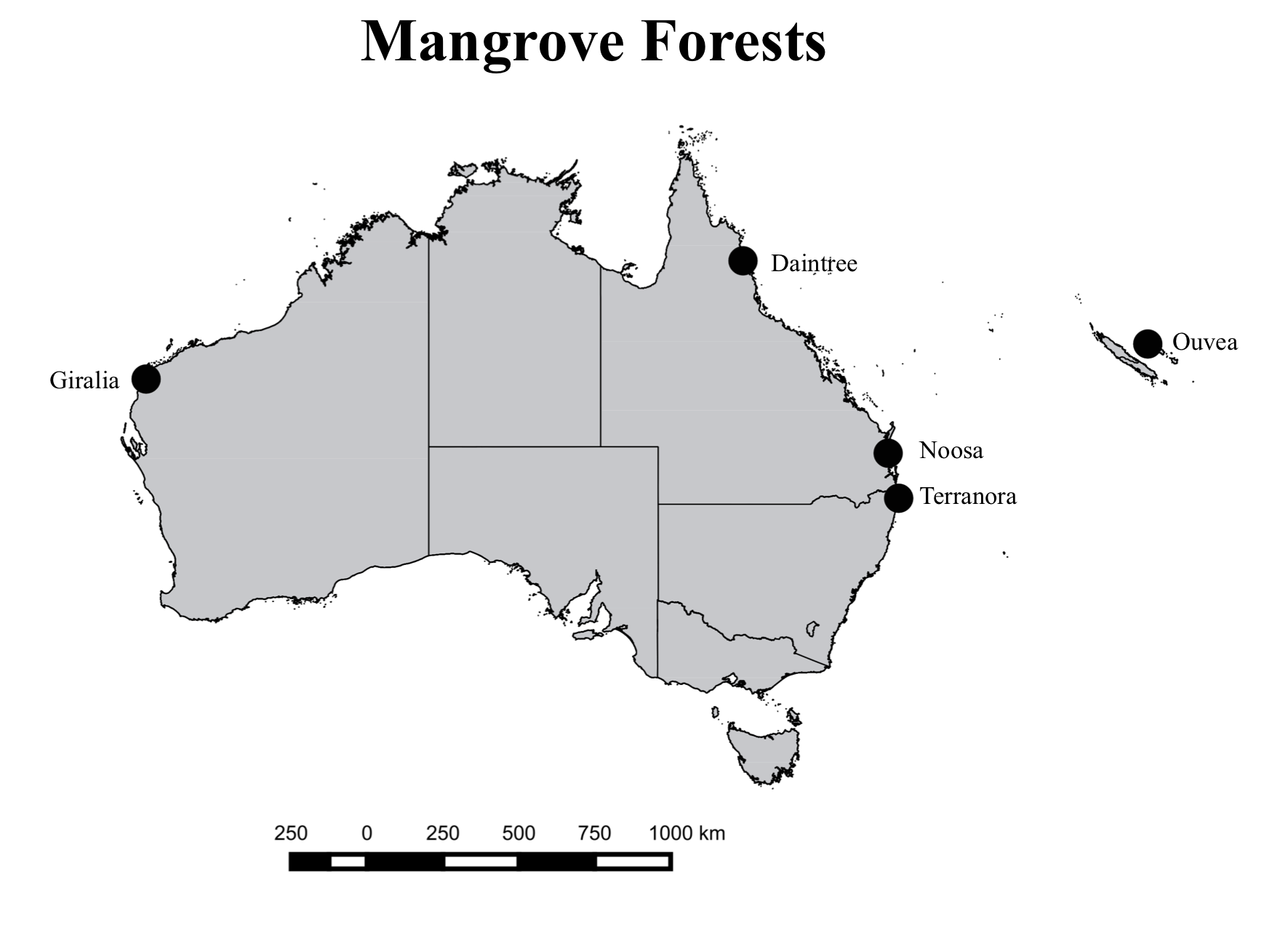

Supplement: S1 Fig — (TIFF) [file pone.0221950.s002.tiff]

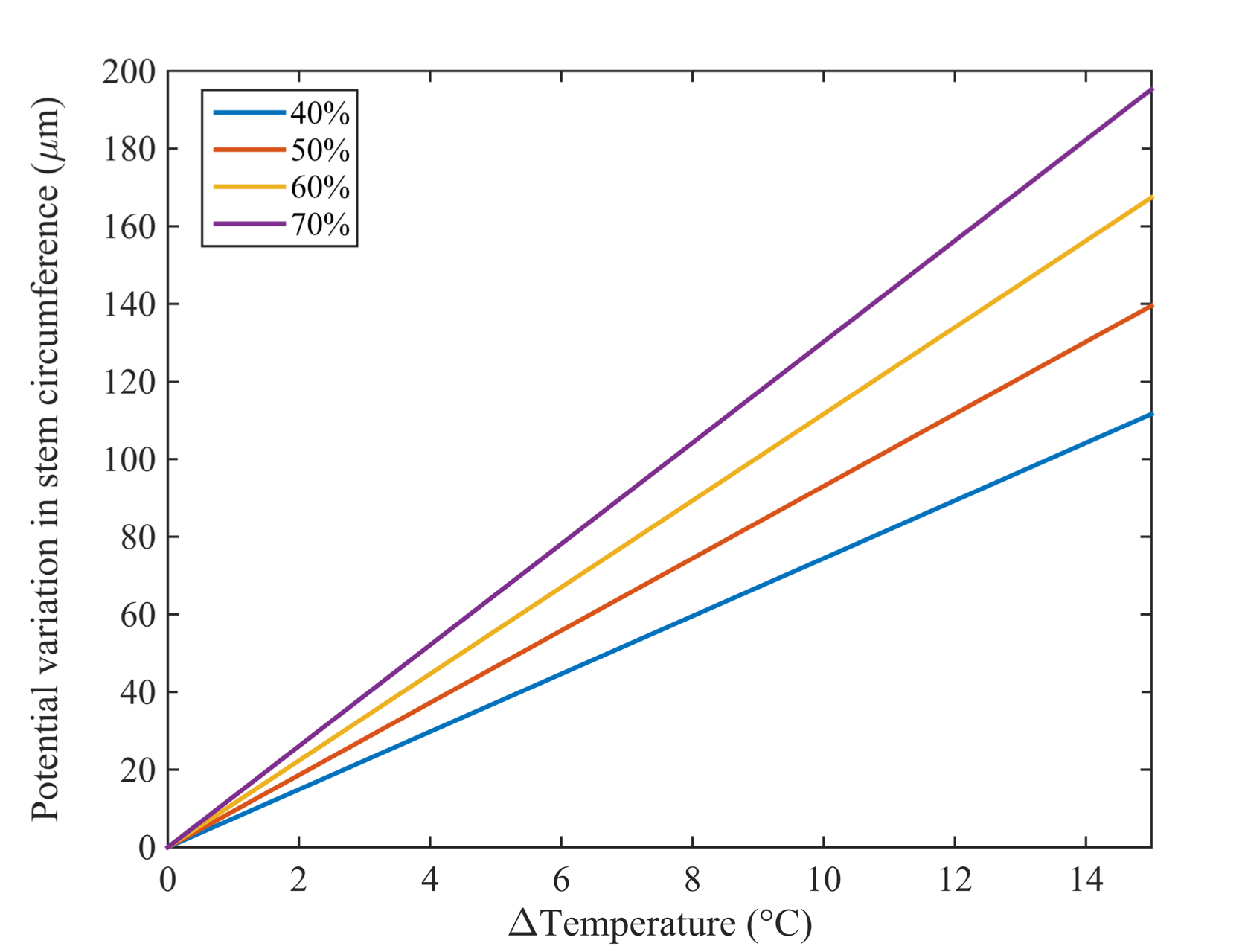

Supplement: S2 Fig — Calculated potential variation in stem circumference with variation in temperature of the stem for a range of stem water contents and for temperature variations of 0.5–15°C. (TIFF) [file pone.0221950.s003.tiff]

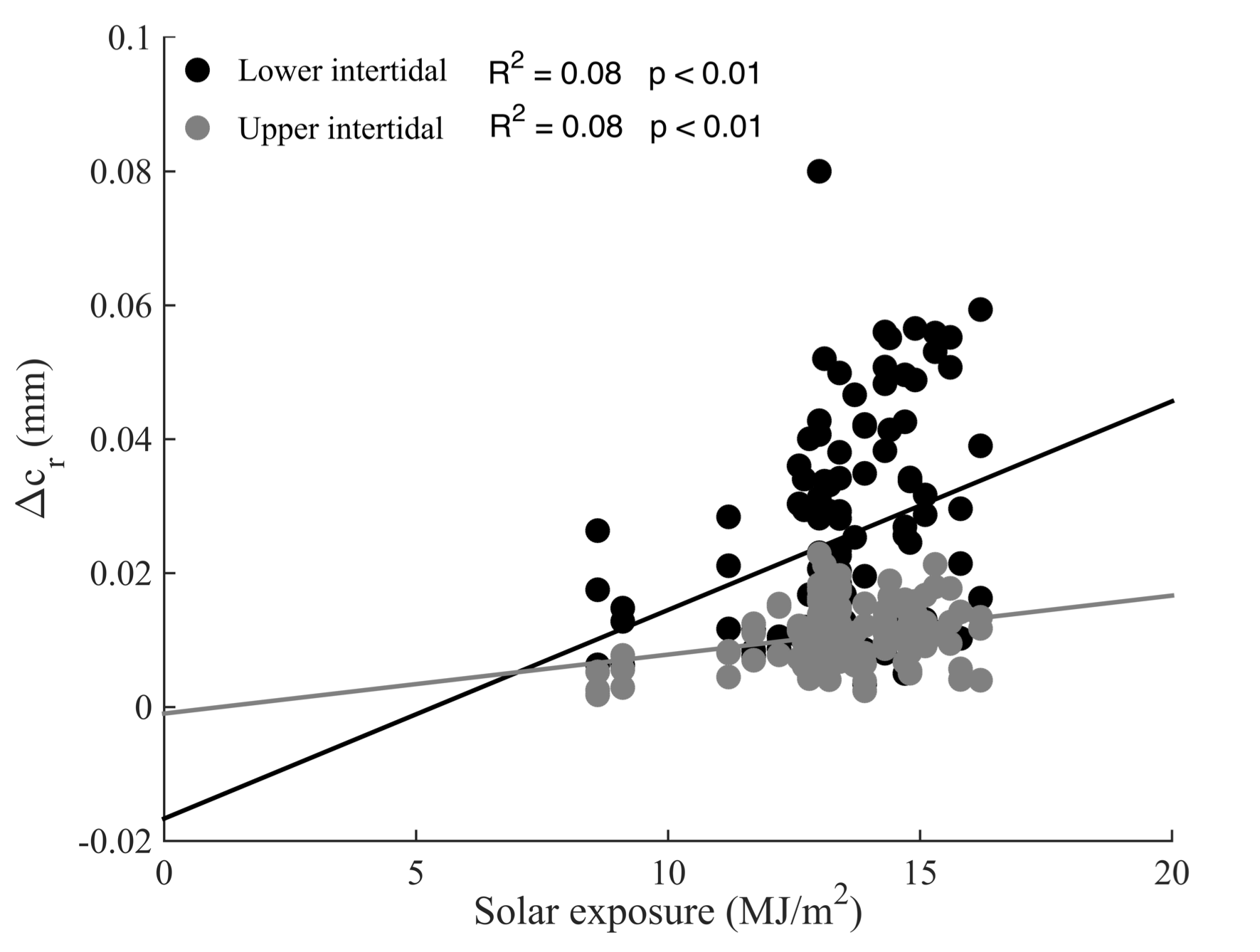

Supplement: S3 Fig — Residual variation (Δcr, mm) versus solar exposure (MJ/m2). (TIFF) [file pone.0221950.s004.tiff]
